# The Chemical Composition and Functional Properties of Essential Oils from Four Species of *Schisandra* Growing Wild in the Qinling Mountains, China

**DOI:** 10.3390/molecules23071645

**Published:** 2018-07-05

**Authors:** Xiaorui Wang, Yan Liu, Yuanyuan Niu, Nongxue Wang, Wei Gu

**Affiliations:** 1 National Engineering Laboratory for Resource Development of Endangered Crude Drugs in Northwest of China, College of Life Sciences, Shaanxi Normal University, Xi’an 710119, China; wangxiaorui@snnu.edu.cn; 2 The Key Laboratory of Medicinal Resources and Natural Pharmaceutical Chemistry, The Ministry of Education, College of Life Sciences, Shaanxi Normal University, Xi’an 710119, China; liuyan68@snnu.edu.cn (Y.L.); n201706016@snnu.edu.cn (Y.N.); wangnongxue@snnu.edu.cn (N.W.); 3 College of Life Sciences, Shaanxi Normal University, Xi’an 710119, China

**Keywords:** *Schisandra*, essential oils, ultrasonic-assisted extraction, antioxidant activity

## Abstract

The aim of this study was to investigate the chemical composition and functional properties of the essential oils from the plants *Schisandra grandiflora* (Wall.) Hook. f. et Thoms, *Schisandra rubriflora* (Franch). Rehd. et Wils., *Schisandra sphenanthera* Rehd. et Wils., and *Schisandra propinqua* (Wall.) Baill var. *sinensis* Oliv. collected in the Qinling Mountains. Under the optimum conditions of the ultrasonic-assisted extraction method, the extraction yields were 7.51% (*S. grandiflora*), 6.91% (*S. rubriflora*), 6.11% (*S. sphenanthera*), and 5.88% (*S. propinqua*). A total of 86 components were identified from four species of *Schisandra* and 16 components were shared among the essential oils of all samples with different contents. However, some components were identified only in a certain plant, for example, *β*-caryophyllen (*S. grandiflora*), *α*-bulnesene (*S. rubriflora*), and *α*-Chamigrene (*S. propinqua*). Terpenoids (sesquiterpenes and oxygenated sesquiterpenes), accounting for 73.87–82.08% of the total compounds, were the main components. Meanwhile, the antioxidant activities of the essential oils were evaluated through three free radical scavenging assays and a reducing power assay, which were related to the contents of the individual bioactive composition. These results provide a phytochemical foundation for the use of four species, and for the further study of the identification of *Schisandra* species.

## 1. Introduction

The genus *Schisandra* comprises about 30 species of woody and deciduous liana and is widely distributed over Southeastern and Central China [1,2,3]. The fresh fruits of *Schisandra* species can be steeped in wine or eaten directly. Most *Schisandra* species are medicinal plants and their dry fruits are widely used as local traditional medicines; however, only two species, *Schisandra sphenanthera* Rehd. et Wils. (*SS*) and *Schisandra chinensis* (Turcz.) Baill., have been officially listed in the *Chinese Pharmacopoeia* for the treatment of coughs and asthma, palpitations and insomnia, night sweats, liver injury and so on, and have also been attributed antiviral and anticancer activity [4,5]. As the genuine origin of *SS*, *the* Qinling Mountains have a wealth of *SS* resources that are high quality and in a wide growing area [6,7]. In comparison, other *Schisandra* species gave attracted little attention because of their limited growing area and narrow range of application. *Schisandra grandiflora* (Wall.) Hook. f. et Thoms. (*SG*), *Schisandra rubriflora* (Franch). Rehd. et Wils. (*SR*) and *Schisandra propinqua* (Wall.) Baill. var. *sinensis* Oliv. (*SP*) are the three species of *Schisandra* that mainly grown in the Qinling Mountains [8]. They were used as herbal medicines against HIV [9,10] and many other diseases [11].

Lignans are considered to be the pharmacologically active components of *Schisandra* species and it many kinds of lignans have been isolated from this genus, such as dibenzocyclooctadiene, dibenzylbutane, and tetrahedrofurane [12,13]. However, up until now, little attention has been paid to the essential oils of four species of *Schisandra* and the antioxidant activity of essential oils. Essential oils are complex mixtures of terpenoids, aromatics, esters, alcohols, and ketones obtained from plant material. The analysis of essential oils is a significant and growing area, as the components of essential oils present in plant materials are increasingly recognized to possess aromatic, antibacterial, insecticidal, and biological properties [14,15,16,17].

The present study was performed to investigate the components of essential oils from four species of *Schisandra* growing in the Qinling Mountains, as well as to understand their antioxidant activities, which were evaluated through the three free radical scavenging assays and the reducing power assay, and this study could provide useful information for the further study of the four medicinal species.

## 2. Results and Discussion

### 2.1. Optimization of the Extraction Yield

**Figure 1:**
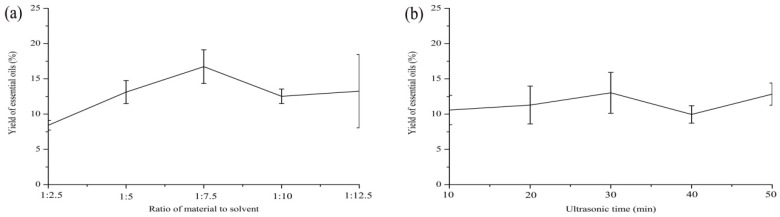

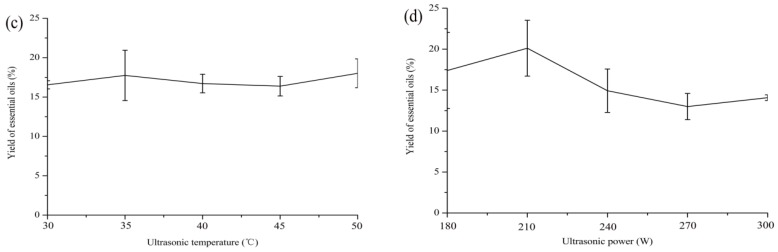
Effect of different parameters on the extraction yield of essential oils from *S. sphenanthera*: (**a**) ratio of material to solvent; (**b**) ultrasonic time; (**c**) ultrasonic temperature; (**d**) ultrasonic power.

It is well known that various parameters play an important role in the optimization of the experimental conditions for the development of the ultrasonic-assisted extraction method. Raw material to solvent ratio, ultrasonic time, ultrasonic temperature, and ultrasonic power are generally considered to be the most important factors that affect the yield (%) of essential oils. Figure 1 shows the experimental results of the single-factor experiment, which is applied to better understand the effects of parameters. As shown in the graph, ultrasonic power and the raw material to solvent ratio were the most significant parameters.

Four independent variables with three variation levels are listed in Table S1. Table S2 shows the experimental conditions and extraction yields for each test. The highest extraction yield was obtained in test 8 at a 1:10 ratio, 30 min, 30 °C, and 240 W. As laid out in the table below, the extraction yields were significantly different when we changed the operating conditions. According to the results of the analysis of variance table (Table S3), we found that the effect of ultrasonic power on essential oils extracted from *SS* was extremely significant but the effects of the other factors were not significant. The factors influenced the yield (%) of essential oils in the following order: ultrasonic power > ultrasonic temperature > ultrasonic time > ratio of material to solvent. In confirmation tests, the content of essential oils extracted from *SS* by the optimum extracting technology reached 6.07%, which was the average value of the triplicate experiments.

### 2.2. Yield of Essential Oil

In this study, we examined the colors and yields (% *v*/*w*) of essential oils from the fruits of four species of *Schisandra*. All the samples studied were fragrant and had different colors: milk white for *SP*, yellow for *SS*, saffron yellow for *SG*, and wheat for *SR*. The yield of fruit essential oil ranged from 5.88% to 7.51% (*v*/*w*). The highest yield of essential oils was *SG* (7.51%), followed by *SR* (6.91%), *SS* (6.11%), and *SP* (5.88%). The yields of essential oils from the fruits of four species far exceeded the yield of the essential oils from the fruits of *SS* obtained from hydrodistillation [18].

The essential oils, which were extracted from aromatic plants, had a wide range of medicinal and industrial applications [19]. According to our literature survey, many factors influenced the yields of essential oils, such as harvest time [20], developmental stage [21], different parts [22,23], and environmental conditions [24]. To reduce the influences of the factors in the present study, we selected the same parts (fruits) of *Schisandra* species, and sampled them at the same time. Consequently, the influences of different parts, harvesting time, and technical parameters were considered negligible. In our study, the three wild *Schisandra* species (*SS*, *SG*, and *SR*) were sampled in Taibai, grown in the same environmental conditions, and *SP* were sampled in Pingli (Table 1). The differences in the yield and composition of essential oils could mainly be attributed to the genotypes of different species of *Schisandra*, but for *SP*, we also need to consider the effect of the environment.

### 2.3. Essential Oils Composition

**Figure 2:**
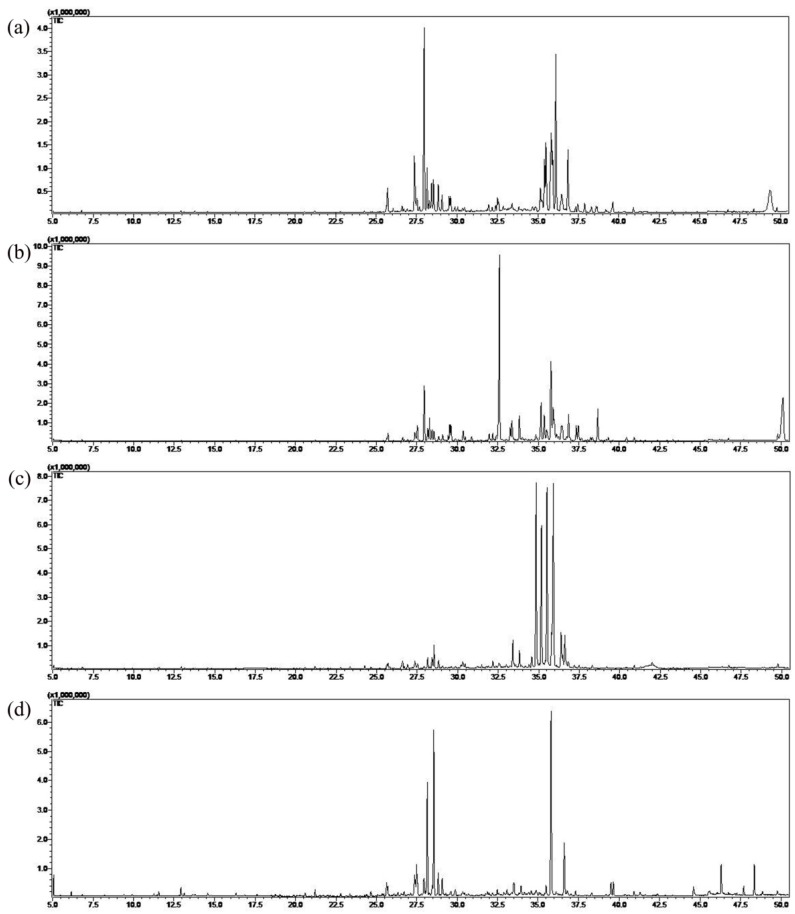
The total ion chromatograms of four species ((**a**) *SG*; (**b**) *SR*; (**c**) *SS*; (**d**) *SP*) in *Schisandra* under GC-MS.

Figure 2 shows a comparative chromatogram of essential oils from the fruits of four species of *Schisandra*, and detailed components are compared in Table S4. The components of essential oils from all samples with low content (<0.1%) are not listed in Table S4. A total of 86 components were identified from all samples, and 16 components were shared among the essential oils of *Schisandra* species, with different contents. The major shared components of essential oils from four species belonged to the sesquiterpenes and oxygenated sesquiterpenes, such as *β*-himachalene and *α*-bisabolol. However, some components were identified only in a certain plant (Table 2). For example, *β*-caryophyllene was a characteristic component of *SG*. *α*-Bulnesene was only found in *SR*. Longiverbenone was only identified in *SS* and *α*-chamigrene only in *SP*.

To evaluate the biodiversity of the essential oils in the four species, the identified components were classified by their chemical structure with the help of literature data and reference components [25]. In Table S4, we list six chemical groups of components in the essential oils from four species of *Schisandra*. The four species had similar chemical groups: sesquiterpenes and oxygenated sesquiterpenes were the principal components, accounting for 30.43–59.64% and 15.82–51.65%, respectively. However, there were many differences in the kinds and numbers of components between the two groups. This situation was caused by the stable genotypes and different levels of gene expression of *Schisandra* species. The results of our study were consistent with previous studies in that sesquiterpenes and oxygenated sesquiterpenes were major chemical groups in the *Schisandra* extracts [26,27].

In this study, the essential oils extracted from the fruits of four species had some identical components to those reported previously in the literature. For instance, *δ*-cadinene, *β*-chamigrene, and *γ*-muurolene were also found in *SS* purchased from Shanghai Pharmacy Co., Ltd., Shanghai, China [18]. *α*-Muurolene, caryophyllene, and *γ*-elemene were also the major components in *SP* purchased from Chinese medicine market, Zunyi, Guizhou, China [28]. However, in previous studies, research into the essential oils in *SG* and *SR* was not mentioned, and our study is the first to research the essential oils of two of the species.

The biological activities of the essential oils are related to the major components. For the essential oils of *SG,* isocaryophyllene and ylangene were the dominant components. Ylangene showed significant inhibitory activities against protein—tyrosine phosphatase 1B [29]. Isocaryophyllene was the precursor of *β*-caryophyllene, which was known for its anti-inflammatory, local anesthetic, and repellent activity against the adult form of *Lasioderma serricorne* [30,31]. Elemol was the major component of *SR*, and as a fragrance ingredient it was used in cosmetics, fine fragrances, shampoos, toilet soaps, and other toiletries as well as in non-cosmetic products [32]. There were three more prominent components of essential oils from the fruits of *SS*. Isospathulenol was the isomer of spathulenol, which had an immune-inhibitory effect on activated lymphocytes [33], longiverbenone was classified as an active toxic compound [34], and the use of cedrenol had similar effects to that of elemol [35]. However, the functions of the dominant components of *SP* were various: *α*-muurolene was the enantiomer of (+)-*δ*-cadinene, which played an important role in the biosynthesis of gossypol [36], and caryophyllene was a metabolite of both plants and fungi, and was also biologically active as an immunosuppressive, cytotoxic, antibacterial, and antifungal agent [37].

### 2.4. Antioxidant Activities of Essential Oils

**Figure 3:**
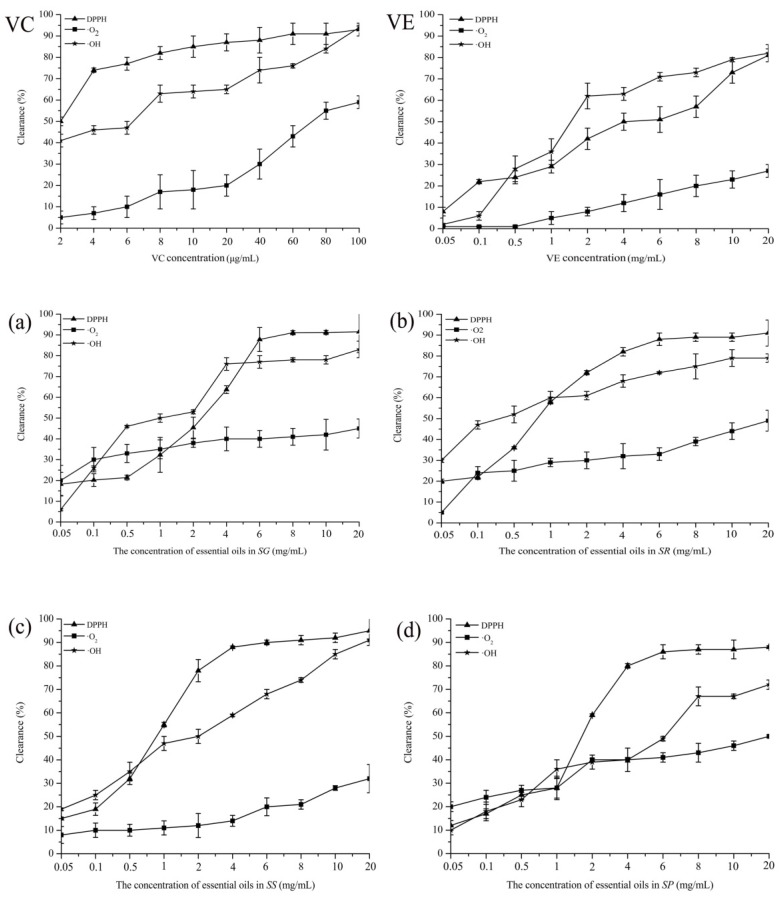
The results of free radical scavenging activities of essential oils from four species ((**a**) *SG*; (**b**) *SR*; (**c**) *SS*; (**d**) *SP*).

Antioxidant activity is a complex process and is the sum of several mechanisms, such as free radical scavenging and reducing capacity [38]. In Figure 3, the essential oils from all four species of *Schisandra* presented high free radical scavenging and low reducing capacity. There were significant differences among the free radical scavenging activities of four species, while it was kept at a constant level with a continuous increase in concentration, and their 50% scavenging capacity (EC_50_) values are listed in Table 3. VC and VE were the positive control.

The scavenging capacities of the essential oils from all samples on DPPH radical were much lower than VC, which achieved a maximum value of 93% when the concentration increased to 100 μg/mL. The capacity of scavenging activity on superoxide anion free radical was in the same descending order (EC_50_): VC > *SP* > *SG* ≈ *SR* > *SS* > VE, different from the results of the DPPH radical scavenging assay. Moreover, the capacity of scavenging activity on hydroxyl free radical was in the same descending order (EC_50_): VC > *SR* > *SG* > VE > *SS* > *SP*.

**Figure 4:**
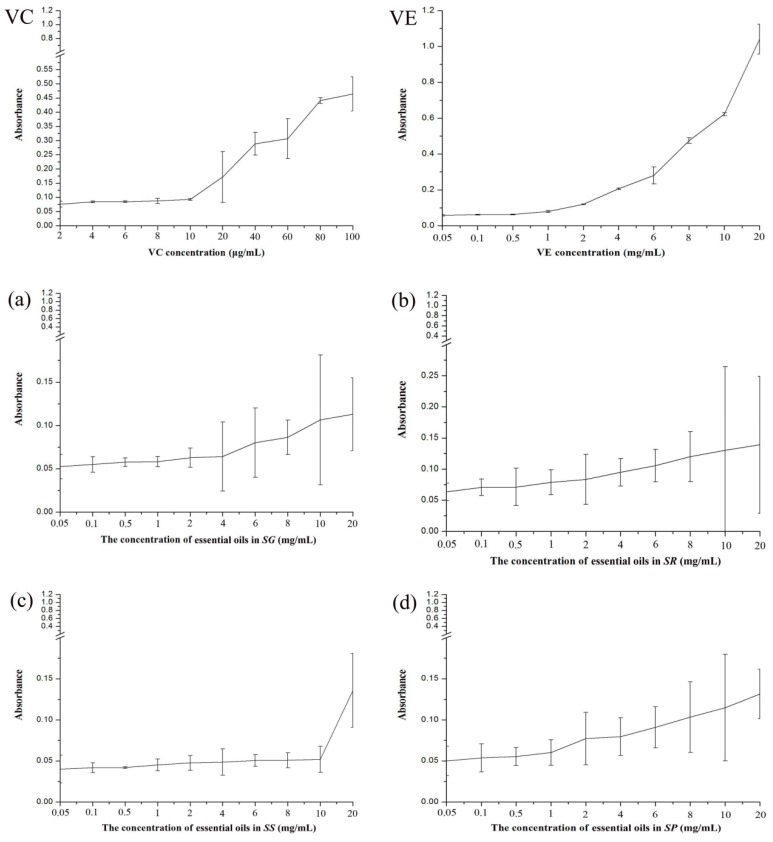
The results of reducing power assay of essential oils from four species ((**a**) *SG*; (**b**) *SR*; (**c**) *SS*; (**d**) *SP*).

The results of the reducing power assay of essential oils from the four species in comparison with VC and VE as the reference antioxidant are presented in Figure 4. The reducing ability increased with increasing oil concentrations and the value of absorbance revealed the reducing power. In Figure 4, the capacities of the reducing power of the essential oils were quite a bit lower than the reference antioxidant.

There were large differences in the free radical scavenging activity of the essential oils from four species of *Schisandra* (Table 3). The free radical scavenging activity of essential oils could be due to their higher content of sesquiterpenes and oxygenated sesquiterpenes components, which were isocaryophyllene, isospathulenol, *α*-muurolene, and longiverbenone according to the GC-MS analysis (Table S4).

## 3. Materials and Methods

### 3.1. Materials and the Extraction of Essential Oils

Plant materials were collected from different regions of the Qinling Mountains (Table 1) and air-dried in the shade at room temperature. All of the samples were identified by Professor Yi Ren (Professor of Plant Taxonomy, Shaanxi Normal University) and a voucher specimen was kept in the College of Life Sciences, Shaanxi Normal University. Every species included at least 10 individuals as one sample. The fruits of all plant materials were comminuted to dried powders separately (screen size 40 meshes) in a pharmaceutical disintegrator, which was used for each extraction to determine the content of the essential oils. Each dried powder sample was extracted with petroleum ether at a designed time, temperature, power and raw material to solvent ratio through the ultrasonic-assisted extraction method [39,40]. The extraction solution was separated from the insoluble residue by centrifugation (1200 rpm for 3 min) and then we collected the supernatant.

### 3.2. Optimization of Essential Oil Extraction

An orthogonal L_9_(3)^4^ test design was used to investigate the optimal extraction conditions of essential oils from *SS* and the extraction experiment was carried out with four factors and three levels in Table S1. The range of each factor level was based on the results of the single-factor experiments. The yield (%) of essential oils from *SS* was the dependent variable, which was calculated as the content of essential oils divided by the dried pre-treated sample weight. Nine experiments were performed in order to estimate the best conditions for the extraction of essential oils, and it was repeated three times to avoid errors.

### 3.3. Analytical Procedures (GC/MS)

Analyses of essential oils were carried out on a Shimadzu (Japan) GC-MS-QP 2010 system using a chromatographic column, Rtx-5ms (30 m × 0.25 mm, 0.25 µm film thickness, 5% diphenyl—95% dimethyl polysiloxane) [41,42]. The oven column temperature was programmed as follows: the initial temperature was 50 °C for 2 min, then increased at a rate of 4 °C/min to 180 °C and held isothermally at 180 °C for 2 min, and finally increased to 240 °C at a rate of 5 °C/min, which was maintained for 2 min. Then, 1.0 µL of the sample was injected at an injection port temperature of 230 °C. The ionizing voltage was 70 eV and the mass spectra were obtained by automatic scanning at *m*/*z* 35–550 amu.

### 3.4. Composition Identification

Through comparing the retention time (RT) and retention indices (RI) with the analysis done under the same temperature and the same chromatographic conditions, the essential oils from four species of *Schisandra* were preliminarily identified. Further identification of components was based on matching their recorded mass spectra with those from the NIST05.LIB and NIST05s.LIB library data (National Institute of Standards and Technology) provided by the software of GC-MS data systems [43]. The relative concentration of the components was calculated by comparing its GC peak area to the total area, were summed from all peaks that were detected (%).

### 3.5. Analyses of Antioxidant Activities

The free radical scavenging capacity of the essential oils was measured using the stable DPPH (2,2-diphenyl-1-picrylhydrazyl) radical, superoxide anion radical (pyrogallol autoxidation), and hydroxyl radical (Fenton reaction), according to the method of Blois 1958 [44], Xu et al. 2013 [45], and Harminder et al. 2010 [46], respectively. Every method was used with a slight modification. The essential oil solutions were diluted to different concentrations (0.05, 0.1, 0.5, 1, 2, 4, 6, 8, 10, and 20 mg/mL) by petroleum ether. VC (Vitamin C) and VE (Vitamin E) were used as standards and were subjected to the same procedure for comparison. The capability to scavenge the free radical was calculated using the following equation: (%) = [*A*_0_ − (*A_i_* − *A_s_*)]/*A*_0_ × 100, where *A*_0_ was the absorbance value of the blank, *A_i_* was the absorbance in the presence of the sample and the free radical, and *A_s_* was the absorbance of the sample without free radical. All determinations were done in triplicate and the concentration of essential oils that reduced the absorption of free radical solution by 50% (EC_50_) was obtained from a linear regression analysis.

Briefly, a solution of DPPH in ethanol was prepared and this solution (100.0 μL) was added to 100.0 μL of essential oil solutions at different concentrations. The mixture was shaken and left to stand for 30 min in the dark at room temperature, and then we measured the absorbance at 517 nm [47].

Three milliliters of Tris-HCL buffer solution (pH 8.2) were incubated in a water bath at 25 °C for 20 min, and this solution was added to 2.0 mL of essential oil solutions at different concentrations. One milliliter of pyrogallol solution (70 mM) was added to the mixture. Absorbance measurements were made at 420 nm after 4 min of reaction at room temperature.

The reaction mixture contained 1.0 mL ferrous sulfate solution (10 mM), 1.5 mL hydrogen peroxide (10% *w*/*v*) solution, and various concentrations of essential oils. After mixing and standing for 10 min, the reaction mixture was added to 2.0 mL salicylic acid (10 mM) solution and then left to stand for 30 min. The absorbance was measured at 510 nm against an appropriate blank solution and all experiments were performed in triplicate.

### 3.6. Reducing Power Assay

The reducing power of essential oils was determined by the method of Oyaizu 1986 [48], with minor modifications. An aliquot of essential oils at various concentrations (0.05, 0.1, 0.5, 1, 2, 4, 6, 8, 10, and 20 mg/mL) was mixed with 2.5 mL potassium ferricyanide (1% *w*/*v*) solution and 2.5 mL phosphate buffer (0.2 M, pH 6.6). The mixture was incubated in a water bath at 50 °C for 20 min, cooled, and then added to 2.5 mL trichloroacetic acid (10% *w*/*v*). The mixture was centrifuged at 3000 rpm for 10 min. The supernatant (2.5 mL) was mixed with 2.0 mL distilled water and 0.5 mL (0.1%) anhydrous iron (ΙΙΙ) chloride (FeCl_3_), and the absorbance was measured at 700 nm using an appropriate blank. VC and VE were used as references. Assays were carried out in triplicate and higher absorbance values indicated stronger reducing power.

### 3.7. Statistical Analysis

The concentrations (%) of chemical constituents in essential oils were used to examine the relationship among four species of *Schisandra*. The antioxidant tests were conducted in triplicate and the results were expressed as mean ± standard deviation (SD). The contents of chemical groups were selected as the clustering variables.

## 4. Conclusions

In this work, we investigated the chemical components and antioxidant activities of the essential oils in four species of *Schisandra* through GC-MS and antioxidant assay. It is clear that the essential oils of all samples are mainly composed of sesquiterpenes and oxygenated sesquiterpenes, which have good biological activity. Moreover, there are obvious differences between the kinds and contents of essential oils from the fruits of four species. Meanwhile, the antioxidant activity of the essential oils is affected by the bioactive composition. The results of our study could be considered the first to provide a direct comparison of the chemical components of the essential oils for these important traditional medicines, and could provide data for the further exploitation of *Schisandra* species.

## Figures and Tables

**Table 1:** A summary of the tested samples.

Sample Name	Sources	Elevation (km)	Longitude	Latitude	Acquisition Time
*S. grandiflora*	Taibai, Baoji, Shaanxi	2.03	107°30.576′	34°01.330′	September 2013
*S. rubriflora*	Taibai, Baoji, Shaanxi	1.95	107°17.705′	33°59.836′	September 2013
*S. sphenanthera*	Taibai, Baoji, Shaanxi	1.23	106°36.396′	32°50.795′	September 2013
*S. propinqua*	Pingli, Ankang, Shaanxi	0.60	109°18.392′	32°23.350′	September 2013

**Table 2:** The particular components of sesquiterpenes and oxygenated sesquiterpenes of four species of *Schisandra*.

Species	Compound Name	RC ^b^ (%) ± SD
*SG*	*β*-Sesquiphellandrene	1.33 ± 0.43
	*β*-Caryophyllene	3.52 ± 0.31
	Isocaryophyllene	12.50 ± 4.36
	*α*-Guaiene	4.51 ± 0.22
	d-nerolidol	1.45 ± 0.19
	Epiglobulol	1.91 ± 1.57
*SR*	*δ*-Elemene	2.13 ± 1.10
	*α*-Caryophyllene	0.34 ± 0.05
	*α*-Ylangene	1.14 ± 0.03
	4,5-dehydro- Isolongifolene	3.73 ± 0.08
	*α*-Bulnesene	2.71 ± 0.71
	*α*-Farnesene	1.31 ± 0.19
	Humulane-1,6-dien-3-ol	0.93 ± 0.03
	Muurolol	0.64 ± 0.05
	Elemol	15.08 ± 0.20
*SS*	Cadinene	0.38 ± 0.08
	*δ*-Cadinene	2.45 ± 0.13
	Germacrene d-4-ol	0.68 ± 0.20
	Cedrenol	10.43 ± 1.06
	Longiverbenone	10.72 ± 1.35
*SP*	*α*-Chamigrene	1.34 ± 0.17

Note SD: standard deviation; b: Relative composition.

**Table 3:** The results for free radical scavenging of four species.

Oxidant	Sample	Regression Equation	EC_50_ (mg/mL) ± SD
DPPH	VC	*y* = 92.180 − 82.651/*x*	1.960 × 10^−3^ ± 0.060
	VE	*y* = 0.018*x*^3^ − 0.707*x*^2^ + 10.178*x* + 17.532	4.368 ± 0.052
	*SG*	*y* = 0.029*x*^3^ − 1.281*x*^2^ + 17.646*x* + 15.944	2.291 ± 0.036
	*SR*	*y* = 0.082*x*^3^ − 2.710*x*^2^ + 24.830*x* + 24.684	1.162 ± 0.054
	*SS*	*y* = 0.099*x*^3^ − 3.242*x*^2^ + 28.974*x* + 20.162	1.180 ± 0.013
	*SP*	*y* = 0.074*x*^3^ − 2.569*x*^2^ + 25.514*x* + 12.019	1.797 ± 0.023
Superoxide anion	VC	*y* = −0.002*x*^2^ + 0.741*x* + 6.553	73.027 × 10^−3^ ± 2.036
	VE	*y* = 0.002*x*^3^ − 0.141*x*^2^ + 3.430*x* + 0.716	44.362 ± 3.046
	*SG*	*y* = 0.019*x*^3^ − 0.618*x*^2^ + 5.528*x* + 27.319	21.625 ± 2.233
	*SR*	*y* = 0.010*x*^3^ − 0.385*x*^2^ + 5.078*x* + 18.510	21.860 ± 3.215
	*SS*	*y* = −0.048*x*^2^ + 2.156*x* + 8.444	25.510 ± 2.357
	*SP*	*y* = 0.021*x*^3^ − 0.690*x*^2^ + 6.909*x* + 22.863	19.257 ± 3.456
Hydroxyl radical	VC	*y* = −0.034*x*^2^ + 1.918*x* + 41.245	5.009 × 10^−3^ ± 0.126
	VE	*y* = 0.073*x*^3^ − 2.442*x*^2^ + 23.292*x* + 10.161	2.174 ± 0.079
	*SG*	*y* = 0.069*x*^3^ − 2.286*x*^2^ + 21.085*x* + 22.634	1.544 ± 0.043
	*SR*	*y* = 0.027*x*^3^ − 0.956*x*^2^ + 10.175*x* + 42.756	0.766 ± 0.032
	*SS*	*y* = 0.021*x*^3^ − 0.851*x*^2^ + 11.812*x* + 26.461	2.376 ± 0.069
	*SP*	*y* = 0.007*x*^3^ − 0.438*x*^2^ + 8.533*x* + 18.063	4.862 ± 0.147

Note SD: standard deviation.

## Supplementary Materials

The following are available online, Table S1: Factors and levels for orthogonal test, Table S2: Results of L9 (34) orthogonal test, Table S3: The table of variance analysis, Table S4: Chemical compounds identified in the volatile oils of four species in *Schisandra*.
