# Supplementary material for: The Chemical Composition and Functional Properties of Essential Oils from Four Species of Schisandra Growing Wild in the Qinling Mountains, China
Source: Molecules. 2018 Jul 5;23(7):1645. doi: 10.3390/molecules23071645 (PMC6100523; doi:10.3390/molecules23071645)
Supplement: Supplementary file 1 [file molecules-23-01645-s001.pdf]

SUPPLEMENTARY MATERIAL

Chemical composition and functional properties of essential oils from four species of *Schisandra*, growing wild in Qinling Mountains, China

Xiaorui Wang[1,3] • Yan Liu[2,3] • Shichao Zhou[2,3] • Wei Gu[1,2,3,*]

[1] *National Engineering Laboratory for Resource Developing of Endangered Chinese Crude Drugs in Northwest of China, Shaanxi Normal University, Xi'an 710119, P.R. China;* [2] *Key Laboratory of Medicinal Resources and Natural Pharmaceutical Chemistry, Ministry of Education, Shaanxi Normal University, Xi'an 710119, P.R. China;* [3] *College of Life Sciences, Shaanxi Normal University, Xi'an 710119, P.R. China*

Corresponding author: College of Life Sciences, Shaanxi Normal University, Xi'an, Shaanxi 710119, P.R. China. E-mail address: weigu@snnu.edu.cn.

Table S1. Factors and levels for orthogonal test.

Level	Factor			
	A (g/mL)	B (min)	C (°C)	D (W)
1	1：5	20	30	180
2	1：7.5	30	35	210
3	1：10	40	40	240

Note A: Ratio of material to solvent; B: Ultrasonic time; C: Ultrasonic temperature; D: Ultrasonic power.

Table S2. Results of L9 (3^4) orthogonal test.

Number	Factor				Extraction rate (%)
	A (g/mL)	B (min)	C (°C)	D (W)	
1	1	1	1	1	5.96±0.33
2	1	2	2	2	3.73±0.22
3	1	3	3	3	3.81±0.15
4	2	1	2	3	5.23±0.45
5	2	2	3	1	5.39±0.18
6	2	3	1	2	4.22±0.30
7	3	1	3	2	2.19±0.22
8	3	2	1	3	6.07±0.32
9	3	3	2	1	3.16±0.19
k1	13.50	13.38	16.25	14.51	
k2	14.84	15.19	12.12	10.14	
k3	11.42	11.19	11.39	15.11	
K1	4.50	4.46	5.42	4.84	
K2	4.95	5.06	4.04	3.38	
K3	3.81	3.73	3.80	5.04	
R	1.14	1.33	1.62	1.66	
Factor sequence	D>C>B>A				
Optimal combination	$A_3B_2C_1D_3$				

Note SD: standard deviation; A: Ratio of material to solvent; B: Ultrasonic time; C: Ultrasonic temperature; D: Ultrasonic power.

Table S3. The table of variance analysis.

Factor	Sum of Squares	Degree of freedom	Mean square	F	Significance
A	7.80	2	3.90	1.36	
B	10.26	2	5.13	1.79	
C	11.86	2	5.93	2.07	
D	16.46	2	8.23	2.87	*
error	51.68		2.87		
sum	5707.91				

Note A: Ratio of material to solvent; B: Ultrasonic time; C: Ultrasonic temperature; D: Ultrasonic power; *: $P < 0.05$.

Table S4. Chemical compounds identified in the volatile oils of four species of *Schisandra*.

No.	Name	Molecular formula	RC [b] (%) ± SD				RI [a]
			SG	*SR*	*SS*	*SP*	
	**Alkanes**		**4.45**	**6.25**	**3.69**	**6.32**	
1	2,4-Dimethylheptane	C_9H_{20}	0.11±0.00	0.50±0.18	0.66±0.18	-	821
2	4-Methyloctane	C_9H_{20}	1.83±0.42	1.09±0.19	0.67±0.58	2.11±1.21	863
3	2,6-Dimethylundecane	$C_{13}H_{28}$	0.25±0.17	0.75±0.25	-	0.75±0.47	1210
4	2,6-Dimethylnonane	$C_{11}H_{24}$	-	-	0.28±0.08	-	1026
5	3,8-Dimethylundecane	$C_{13}H_{28}$	-	1.91±0.53	0.44±0.08	-	1227
6	6-ethyl-2-methyl octane	$C_{11}H_{24}$	-	-	-	0.73±0.23	1037
7	1-Phenyl-1-methylbutane	$C_{11}H_{16}$	-	-	-	0.26±0.07	1098
8	2,4,6-Trimethyloctane	$C_{11}H_{24}$	1.29±0.31	2.00±1.19	1.20±1.08	1.73±1.04	1058
9	2,7,10-Trimethyldodecane	$C_{15}H_{32}$	0.97±0.42	-	0.28±0.01	0.74±0.54	1368
10	6-Methyltridecane	$C_{14}H_{30}$	-	-	0.16±0.00	-	1316
	**Ketone**		**1.62**	**-**	**0.85**	**-**	
11	2-Benzyl-3-isopropyl-cyclopentanone	$C_{15}H_{20}O$	1.62±0.08	-	-	-	1808
12	*β*-Ionone	$C_{13}H_{20}O$	-	-	0.63±0.00	-	1485
13	Phenacyl acetate	$C_{11}H_{10}O_3$	-	-	0.22±0.07	-	1541
	**Sesquiterpenes**		**59.64**	**42.09**	**30.43**	**52.41**	
14	*α*-Santalene	$C_{15}H_{24}$	-	-	8.32±0.01	0.58±0.33	1211
15	Cuparene	$C_{15}H_{24}$	-	-	0.58±0.08	0.25±0.00	1556
16	*α*-Bisabolene	$C_{15}H_{24}$	-	0.34±0.16	0.40±0.31	0.55±0.17	1443
17	Cadinene	$C_{15}H_{24}$	-	-	0.38±0.08	-	1440
18	*α*-Muurolene	$C_{15}H_{24}$	-	-	0.50±0.07	11.05±2.11	1497
19	*α*-Amorphene	$C_{15}H_{24}$	-	1.27±0.20	0.55±0.20	0.31±0.05	1482

No.	Name	Molecular formula	RC [b] (%) ± SD				RI [a]
			SG	*SR*	*SS*	*SP*	
20	Ylangene	$C_{15}H_{24}$	10.81±1.45	3.90±0.63	2.60±0.06	1.90±0.10	1407
21	*β*-Himachalene	$C_{15}H_{24}$	2.18±0.09	0.98±0.59	2.81±0.06	2.12±0.50	1547
22	*δ*-Elemene	$C_{15}H_{24}$	-	2.13±1.10	-	-	1377
23	*β*-Bisabolene	$C_{15}H_{24}$	0.25±0.00	0.82±0.45	-	0.12±0.00	1506
24	*β*-Sesquiphellandrene	$C_{15}H_{24}$	1.33±0.43	-	-	-	1525
25	*γ*-Muurolene	$C_{15}H_{24}$	0.63±0.00	-	2.35±0.06	-	1474
26	Isosativene	$C_{15}H_{24}$	-	0.33±0.12	-	0.36±0.05	1417
27	*α*-Cedrene	$C_{15}H_{24}$	0.20±0.00	-	0.28±0.19	-	1408
28	*γ*-Cadinene	$C_{15}H_{24}$	-	-	2.15±0.27	1.77±0.28	1435
29	*δ*-Cadinene	$C_{15}H_{24}$	-	-	2.45±0.13	-	1519
30	*α*-Longipinene	$C_{15}H_{24}$	-	0.50±0.13	0.15±0.02	7.79±1.62	1334
31	*α*-Bergamotene	$C_{15}H_{24}$	1.08±0.24	0.58±0.38	0.58±0.05	0.39±0.19	1433
32	*α*-Caryophyllene	$C_{15}H_{24}$	-	0.34±0.05	-	-	1452
33	Germacrene D	$C_{15}H_{24}$	1.25±0.39	0.82±0.02	1.29±0.13	0.79±0.13	1515
34	*α*-Ylangene	$C_{15}H_{24}$	-	1.14±0.03	-	-	1373
35	Elixene	$C_{15}H_{24}$	2.19±1.59	0.50±0.03	-	-	1445
36	4,5-dehydro- Isolongifolene	$C_{15}H_{22}$	-	3.73±0.08	-	-	1393
37	Caryophyllene	$C_{15}H_{24}$	3.11±0.11	4.00±1.14	2.32±0.43	13.88±1.17	1494
38	*γ*-Elemene	$C_{15}H_{24}$	-	1.81±0.03	-	2.26±0.46	1465
39	*δ*-Guaiene	$C_{15}H_{24}$	4.68±0.15	2.10±0.28	0.93±0.11	4.25±0.76	1505
40	Muurolene	$C_{15}H_{24}$	7.49±0.18	7.45±0.12	-	-	1469
41	*β*-Chamigrene	$C_{15}H_{24}$	-	2.63±0.26	0.73±0.46	0.19±0.00	1478
42	*β*-Caryophyllene	$C_{15}H_{24}$	3.52±0.31	-	-	-	1423
43	*α*-Bulnesene	$C_{15}H_{24}$	-	2.71±0.71	-	-	1508

No.	Name	Molecular formula	RC [b] (%) ± SD				RI [a]
			SG	*SR*	*SS*	*SP*	
44	α-Farnesene	$C_{15}H_{24}$	-	1.31±0.19	-	-	1509
45	Isocaryophyllene	$C_{15}H_{24}$	12.50±4.36	-	-	-	1411
46	α-Humulene	$C_{15}H_{24}$	3.25±1.18	0.89±0.01	-	1.15±0.11	1579
47	α-Chamigrene	$C_{15}H_{24}$	-	-	-	1.34±0.17	1514
48	α-Guaiene	$C_{15}H_{24}$	4.51±0.22	-	-	-	1413
49	Germacrene B	$C_{15}H_{24}$	-	1.13±0.30	0.69±0.06	0.99±0.21	1431
50	β-Santalene	$C_{15}H_{24}$	0.66±0.35	0.68±0.13	0.37±0.19	0.37±0.11	1425
	**Oxygenated sesquiterpenes**		**15.82**	**37.30**	**51.65**	**22.70**	
51	3,7,11-Trimethyl-2,6,10-dodecatrien-1-ol	$C_{15}H_{26}O$	1.59±0.31	2.09±0.57	0.61±0.74	0.58±0.07	1695
52	δ-Cadinol	$C_{15}H_{26}O$	-	1.53±0.48	0.75±0.03	0.45±0.04	1652
53	Nerolidol	$C_{15}H_{26}O$	2.58±0.92	2.47±0.56	0.83±0.37	4.59±1.22	1564
54	Humulane-1,6-dien-3-ol	$C_{15}H_{26}O$	-	0.93±0.03	-	-	1619
55	Germacrene D-4-ol	$C_{15}H_{26}O$	-	-	0.68±0.20	-	1569
56	D-nerolidol	$C_{15}H_{26}O$	1.45±0.19	-	-	-	1532
57	α-Cadinol	$C_{15}H_{26}O$	0.80±0.13	-	0.21±0.02	-	1650
58	Farnesyl alcohol	$C_{15}H_{26}O$	-	1.58±0.34	0.29±0.04	4.30±2.04	1713
59	Muurolol	$C_{15}H_{26}O$	-	0.64±0.05	-	-	1644
60	trans-Nerolidol	$C_{15}H_{26}O$	0.63±0.06	-	-	1.98±0.36	1551
61	Elemol	$C_{15}H_{26}O$	-	15.08±0.20	-	-	1537
62	Farnesol	$C_{15}H_{26}O$	0.17±0.00	-	0.27±0.15	0.52±0.20	1416
63	α-Bisabolol	$C_{15}H_{26}O$	1.61±0.48	0.71±0.35	1.50±1.14	1.93±0.60	1680
64	α-Santalol	$C_{15}H_{24}O$	-	2.08±0.04	4.29±5.33	-	1454
65	trans-Farnesol	$C_{15}H_{26}O$	1.55±1.84	-	0.25±0.12	0.43±0.24	1722
66	Spathulenol	$C_{15}H_{24}O$	0.40±0.00	2.65±0.05	2.98±0.28	1.21±0.10	1536

No.	Name	Molecular formula	RC [b] (%) ± SD				RI [a]
			SG	*SR*	*SS*	*SP*	
67	Cedrenol	$C_{15}H_{24}O$	-	-	10.43±1.06	-	1604
68	Longiverbenone	$C_{15}H_{22}O$	-	-	10.72±1.35	-	1651
69	*α*-Bisabolene epoxide	$C_{15}H_{24}O$	-	0.18±0.00	0.12±0.00	-	1531
70	Isospathulenol	$C_{15}H_{24}O$	-	2.98±0.10	11.37±0.92	1.08±0.01	1582
71	Cedr-8-en-15-ol	$C_{15}H_{26}O_3$	1.07±0.22	-	-	0.73±0.09	1646
72	Epiglobulol	$C_{15}H_{24}O$	1.91±1.57	-	-	-	1530
73	Caryophyllene epoxide	$C_{15}H_{24}O$	-	1.62±0.04	0.44±0.01	0.76±0.06	1507
74	*β*-Santalol	$C_{15}H_{24}O$	1.18±0.91	0.50±0.28	0.46±0.08	0.89±0.61	1724
75	Ledene oxide	$C_{15}H_{24}O$	-	1.25±1.54	0.41±0.00	1.01±0.11	2062
76	Santalol	$C_{15}H_{24}O$	0.88±0.21	1.01±0.36	5.04±4.51	2.24±0.18	1617
	**Esters**		**2.51**	**1.73**	**3.42**	**2.74**	
77	Oxalic acid, allyl hexadecyl ester	$C_{21}H_{38}O_4$	-	-	0.78±0.38	-	2342
78	Oxalic acid, cyclobutyl tridecyl ester	$C_{19}H_{34}O_4$	1.38±0.70	1.73±1.47	1.00±0.21	1.92±0.62	2179
79	Allethrin	$C_{19}H_{26}O_3$	0.57±0.41	-	0.75±0.60	-	2065
80	Oxalic acid, cyclobutyl tetradecyl ester	$C_{20}H_{36}O_4$	-	-	0.89±0.62	0.82±0.10	2217
81	Ferulic acid methyl ester	$C_{11}H_{12}O_4$	0.56±0.00	-	-	-	1855
	**Alcohols**		**1.85**	**0.40**	**0.82**	**2.36**	
82	4-Methylcholesta-8,24-dien-3-ol	$C_{28}H_{46}O$	-	-	0.51±0.29	0.89±0.06	3115
83	3,4-Dimethyl-5-hexen-3-ol	$C_8H_{16}O$	-	-	-	0.30±0.06	993
84	3-Butyn-1-ol	C_4H_6O	1.85±0.24	-	0.31±0.68	0.23±0.41	660
85	13-Heptadecyn-1-ol	$C_{17}H_{32}O_2$	-	0.40±0.42	-	0.61±0.29	1784
86	3,3-Dimethylcyclohexanol	$C_8H_{16}O$	-	-	-	0.33±0.08	1392
**Total identified (%)**			**85.89**	**87.77**	**90.86**	**86.53**	

Note SD: standard deviation; a: Retention index on RTX-5MS capillary column; b: Relative composition; –: Not detected.
